# Triclocarban and Health: the Jury Is Still Out

**DOI:** 10.1128/mSphere.00239-16

**Published:** 2016-11-02

**Authors:** Rebekah C. Kennedy, Paul D. Terry, Jiangang Chen

**Affiliations:** a Comparative and Experimental Medicine Graduate Program, The University of Tennessee, Knoxville, Tennessee, USA; b Department of Public Health, University of Tennessee, Knoxville, Tennessee, USA; c Department of Medicine, Graduate School of Medicine, The University of Tennessee, Knoxville, Tennessee, USA; Arizona State University

## LETTER

In a recently published report, Poole et al. utilized a crossover design to determine the effect of nonprescription antimicrobial exposure on the human microbiome (1). After an initial washout phase, participants were provided either products that contained TCS or products without TCS for use over a 4-month period, followed by a switch to the opposite exposure regimen for another 4 months. Typically, in the literature, “TCS” refers to the nonprescription antimicrobial triclosan. In Poole’s article, however, “TCS” was defined as an acronym for triclosan as well as an additional nonprescription antimicrobial, triclocarban. The authors could be confident that triclosan exposure occurred during the active exposure phases as the urine concentration was monitored. However, triclocarban levels were not measured, limiting assumptions regarding this chemical. Even so, within the Importance section of the abstract, it was concluded that the normal use of both triclocarban and triclosan has little influence on oral or gut microbiota or certain metabolic biomarkers. It is concerning that such a broad conclusion regarding the safety of triclocarban exposure to the human microbiome was made without a firm understanding of whether exposure even occurred at all. Although the Discussion section does provide a number of limitations, many readers may only read the abstract of the article. If so, the authors’ conclusion could be misleading and potentially provide a false sense of security regarding the safety of triclocarban-containing product use on microbiome health without adequate data to support these claims.
